# Specific immunotherapy plus Clostridium butyricum alleviates ulcerative colitis in patients with food allergy

**DOI:** 10.1038/srep25587

**Published:** 2016-05-11

**Authors:** B Bin Lan, Fan Yang, Dong Lu, Zhenlv Lin

**Affiliations:** 1 Department of Gastroenterological Surgery, The First Affiliated Hospital, Fujian Medical University, Fuzhou 350005, Fujian, China; 2 Department of Gastroenterology, The First Affiliated Hospital, Fujian Medical University, Fuzhou 350005, Fujian, China; 3 Department of Emergency Surgery, The First Affiliated Hospital, Fujian Medical University, Fuzhou 350005, Fujian, China

## Abstract

The aberrant T cell activation plays an important role in the pathogenesis of intestinal inflammation, such as ulcerative colitis (UC). *C. butyricum* (Cb) is a probiotic and has been employed in the treatment of immune diseases. This study tests a hypothesis that specific immunotherapy (SIT) plus oral Cb (an over-the-counter probiotic) alleviates the UC symptoms. In this study, we conducted a randomized, double-blind, clinical study at our hospital. A total of 80 patients with relapsing-remitting ulcerative colitis and high levels of specific IgE antibody was randomly divided into 4 groups, and were treated with SIT or/and Cb, or placebo, respectively for 1 year. The results showed that a food antigen-specific Th2 polarization immune response was observed in UC patients with food allergy (FA). The frequency of regulatory B cells was significantly less in UC patients with FA as compared with healthy subjects. The UC patients with FA were treated with SIT and Cb showed significant amelioration of UC clinical symptoms, reduction of using UC-control medicines, and suppression of the skewed Th2 polarization, which did not occur in those treated with either SIT alone, or Cb alone, or placebo. In conclusion, combination of SIT and Cb efficiently alleviates a fraction of UC patients.

Inflammatory bowel disease (IBD) is a group of inflammatory conditions of the colon and small intestine, including two major types, Crohn’s disease (CD) and ulcerative colitis (UC). UC mainly affects the colon, while CD can affect the whole digestive tract^1^. IBD can be one of the following types, collagenous colitis, lymphocytic colitis, diversion colitis, Behçet’s disease and indeterminate colitis. However, no disease specific markers are currently known in the blood, enabling the reliable separation of CD and UC patients. The pathogenesis of IBD is less understood currently.

Investigators intend to consider IBD as a complex disease which arises as a result of the interaction of environmental and genetic factors. Published data indicate that alterations to internal bacteria may contribute to the pathogenesis of IBD^2^. Decrease in Firmicutes and Bacteroidetes IBD has been noted in 30–50% IBD patients, which may disturb the biodiversity of commensal bacteria in the intestinal tract. A portion of IBD patients reported that they were prescribed antibiotics in the last 2–5 years before the IBD symptoms occurred^3^. Food allergy (FA) may be another possible cause of IBD^4^ as mast cells can be sensitized by the antigen-specific IgE. Re-exposure to specific antigens can activate mast cells and induce mast cells to release proinflammatory cytokines and some other chemical mediators such as tumor necrosis factor-α, which are sufficient to induce intestinal inflammation. Since the antigen-specific immunotherapy (SIT) is an effective remedy for the treatment of allergic disorders, we hypothesize that SIT may alleviate the clinical symptoms of IBD. On the other hand, based on the feature of improving immunity^5^, probiotics are employed in the treatment of IBD^6^. Thus, in this study, we specifically treated a group of UC patients with FA (hereinafter, FA-UC) with SIT and probiotics. We observed that although treating with either SIT alone or probiotics alone did not show an apparent therapeutic effect on UC, treating with both SIT and probiotics significantly improved the clinical symptoms of UC.

## Results

### Assessment of food antigen specific immune response in UC patients

**Figure 1:**
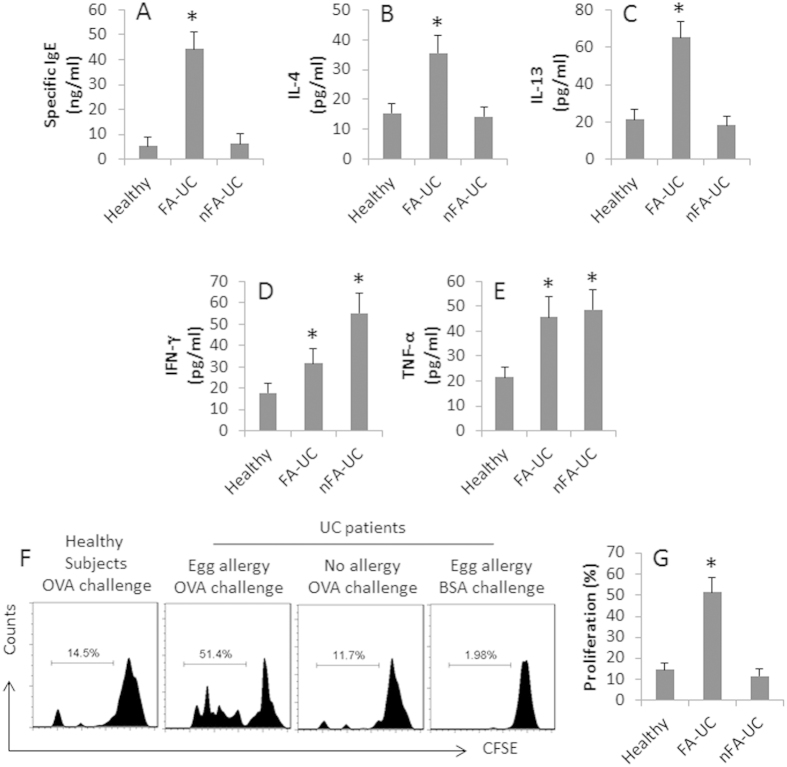
Assessment of Th2 responses in UC patients. The peripheral blood samples from 80 UC patients with FA history (FA-UC), 72 UC patients without FA-history (nFA-UC) and 20 healthy subjects were collected as indicated in the figure. The samples from individual subjects were processed separately. A-E, PBMCs were isolated from the blood samples and cultured in the presence of the sensitized food antigens (5 μg/ml; the type of food antigens was determined by SPT) for 3 days. The supernatant was collected at the end of the culture and analyzed by ELISA. The bars indicate the serum levels of OVA-specific IgE (**A**), IL-4 (**B**), IL-13 (**C**), IFN-γ (D) and TNF-α (**E**). (**F**) A portion of the blood samples was processed for CD4^+^ T cell and DC isolation. The CD4^+^ T cells (labeled with CFSE; 10^5^ cells/well) and DCs (2 × 10^4^ cells/well) were cocultured for 3 days in the presence of OVA (5 μg/ml) or BSA (5 μg/ml) and PMA (20 ng/ml). The histograms indicate the frequency of proliferating UC4^+^ T cells, which were summarized in panel G.

To investigate a potential relationship between IBD and food antigens, we collected the peripheral blood from 80 UC patients with FA history (FA-UC patients); 72 UC patients without FA history (NFA-UC patients) and 20 healthy subjects. The PBMCs were challenged with specific food antigens in the culture for 3 days; the supernatant was analyzed by ELISA. The results showed that the specific IgE was detected in both groups of UC patients, which was significantly higher in FA-UC patients than in nFA-UC patients. Although the healthy subjects did not have the FA history, low levels of specific IgE were also detected, which was compatible with that in nFA-UC patients (Fig. 1A).

We also analyzed the representative cytokines of CD4^+^ T cells in the culture supernatant by ELISA. The results showed that the levels of IL-4 (Fig. 1B) and IL-13 (Fig. 1C) were higher in UC patients with FA than those UC patients without allergy. The levels of IFN-γ (Fig. 1D) and TNF-α (Fig. 1E) were higher in those nFA-UC patients than those FA-UC patients. The specific CD4^+^ T cells were detected in the FA-UC patients (Fig. 1F,G). After challenge with OVA (the specific antigen) in the culture, CD4^+^ T cells from FA-UC produced higher levels of IL-4 than CD4^+^ T cells from nFA-UC patients and healthy subjects, while the levels of IFN-γ were uniquely higher in CD4^+^ T cell culture of nFA-UC (Fig. 1G). The results indicate that a specific immune response exists in the FA-UC patients. The results indicate the FA-UC patients have a skewed antigen-specific Th2 response in the body.

### The frequency of regulatory B cells is lower in UC patients with FA

**Figure 2:**
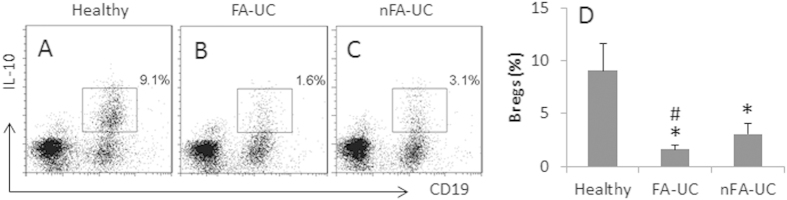
UC patients with FA have less peripheral Bregs. Blood samples were the same as Fig. 1. PBMCs were isolated first and analyzed by flow cytometry. (**A**–**C)** The dot plots indicate the frequency of CD19^+^ IL-10^+^ Bregs. (**D**) The bars indicate the summarized data of (**A**–**C**). Data of bars are presented as mean ± SD. *p < 0.01, compared with healthy subjects. ^#^p < 0.01, compared with nFA-UC patients.

We then analyzed the regulatory B cells (Breg) in the peripheral blood. With the same batch of samples of Fig. 1, we assessed the frequency of IL-10^+^ B cells (Breg) in the PBMC by flow cytometry. The results showed that, as compared with healthy subjects, the frequency of Bregs was lower in FA-UC patients. In the nFA-UC patients, although the frequency of Bregs was lower than healthy subjects, it did not reach the significant levels (Fig. 2).

### Combination of specific immunotherapy (SIT) and *C. butyricum* improves UC

**Figure 3:**
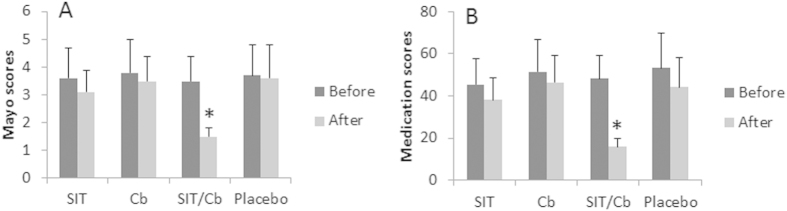
Mayo scores of FA-UC patients after treating with SIT or/and Cb. Mayo scores and medication scores were recorded for each patient before and after the treatment with SIT or/and Cb, or placebo, respectively. The bars indicate the Mayo scores (**A**) and medication scores (**B**) (Mean ± SD). *p < 0.01, compared with the data of before treatment. Number of patients: SIT = 19; Cb = 18; SIT/Cb = 23; placebo = 20.

The FA-UC patients were randomly divided into four groups and treated with SIT or/and *C. butyricum* (Cb), or placebo, respectively, for 12 months. The design was double blind to avoid the observer bias. As shown by the truncated Mayo scores, Both of the SIT group and Cb group showed UC symptom improvement somehow, but did not reach the significant value; the SIT/Cb group showed marked improvement in UC symptoms. No statistical changes were observed in the placebo group (Fig. 3A). When necessary, the patients were allowed to take regular medicines (mesalamine or/and prednisone; Table 1) during the study period. The results showed that the combination with SIT/Cb significantly reduced the medication scores, while patients treated with either SIT alone, or Cb alone, or placebo, did not show statistical changes in their medication scores (Fig. 3B). The results indicate that the combination of SIT and Cb can markedly improve the clinical symptoms of UC.

### Combination of SIT and Cb improves the immunity of UC

**Figure 4:**
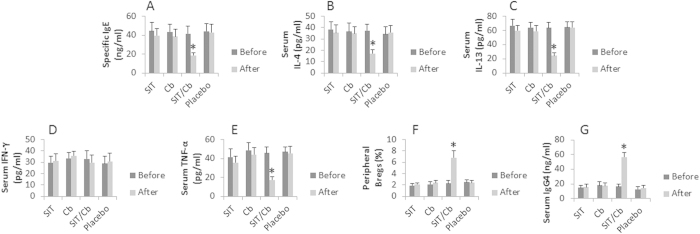
Representative immune parameter changes in UC patients. FA-UC patients were randomly divided into 4 groups. Peripheral blood samples were obtained from each patient before and after the indicated treatment (on the X axis of each subpanel). The sera were analyzed by ELISA. PBMCs were isolated and analyzed by flow cytometry. (**A–E**) The bars indicate the serum levels of specific IgE (**A**), IL-4 (**B**), IL-13 (**C**), IFN-γ (**D**) and TNF-α (**E**). (**F**) The bars indicate the frequency of Bregs (**F**). (**G**) The bars indicate the serum levels of specific IgG. Data of bars are presented as mean ± SD. *p < 0.01, compared with the placebo group. Samples from individual patients were analyzed separately.

The effect of SIT or/and Cb administration on serum specific IgE, IL-4, IL-13, IFN-γ, TNF-α, Bregs and serum specific IgG4 of UC patients with FA was evaluated before and after the treatment. As shown by Fig. 4, the treatment with SIT/Cb significantly decreased the serum levels of specific IgE, IL-4, IL-13 and TNF-α, and increased the frequency of peripheral Bregs and serum IgG4. The serum levels of IFN-γ were only slightly altered. Those treated with either SIT alone or Cb alone, and placebo did not show improvement in the immune parameters. The results indicate that the treatment with SIT/Cb can markedly improve the immunity of FA-UC patients.

## Discussion

Current therapeutic remedies for IBD mostly focus on the symptom control. Two major categories of medicines, including 5-aminosalicylate and steroids, are commonly prescribed for IBD patients. The role of 5-aminosalicylate is to inhibit inflammation. The purpose of using steroids is to suppress immune response. Although these medicines are quite effective for control IBD clinical symptoms, once stopping medication, the IBD clinical symptoms relapse sooner or later. For the purpose of seeking an effective remedy for IBD, the present study treated a group of FA-UC patients with SIT and Cb. Although either SIT alone or Cb alone did not show an apparent therapeutic effect on the suppression of UC symptoms, a combination of SIT and Cb resulted in much better results on alleviating UC.

In this study, we observed a skewed Th2 response in FA-UC patients. They had positive skin test results for one or more food antigens. This is the relative reliable evidence to diagnose FA. The diagnosis is supported by the presence of food antigen-specific IgE and the presence of food antigen-specific Th2 cells in the peripheral blood system. Thus, the FA in these UC patients was active. The relation between IBD and FA has been discussed in the literature. Such as Judaki *et al.* indicate that there is a significant relationship between UC and cow milk^7^. UC patients who are allergic to dairy products and the use of dairy products can increase the severity of UC^7^. Kawaguchi *et al.* indicate that the serum levels of food antigen-specific antibodies were significantly higher in IBD patients than in healthy subjects^8^. For those have an apparent FA history IBD patients, to avoid taking the offending foods may be one way to avoid IBD attack. But in the case of the FA is at the sub-clinical status, it is better to screen the FA status in IBD patients to find the offending antigens to eliminate those foods from their diets.

SIT is a specific remedy for the treatment of allergic diseases recommended by the World Health Organization^9^, and is extensively used in the treatment of allergic diseases. We also used SIT to treat the FA-UC patients in this study. However, treating with SIT alone did not get satisfactory outcomes in the alleviation of the UC clinical symptoms. Others also observed similar phenomenon. Such as Roger *et al.* treated a group of patients with allergic rhinitis and found that the SIT had no significant effect on concentrations of total IgE, specific IgE or Th2 cytokines, although satisfactory relief of allergic rhinitis symptoms was declared by most patients in the period of the treatment^10^. Glover *et al.* found that no effect of SIT on asthma^11^.

It got much better results on alleviating the UC clinical symptoms when we treated FA-UC patients with both SIT and Cb. To treat IBD with probiotics has been reported. Rembacken *et al.* suggest that treatment with a non-pathogenic *E. coli* has an equivalent effect to mesalazine in maintaining remission of ulcerative colitis^12^. Bibiloni *et al.* treated UC patients with probiotics VSL#3 and resulted in 77% remission^13^. However, no apparent alleviation of the UC clinical symptoms was observed in those treated with Cb alone as shown by the present data. The difference between our data and previous data may be because we used different probiotics. Others also found that administration of *L. johnsonii* LA1 did not have a sufficient effect to prevent the endoscopic recurrence of CD^14^.

After treatment with SIT/Cb, we observed a regulatory effect on the skewed Th2 response and increase in peripheral Bregs in the FA-UC patients. This is in line with a recent report. Shi *et al.* observed that the combination of SIT and Cb inhibited the FA-induced intestinal Th2 inflammation in a mouse model, in which the antigen-specific Bregs were increased^15^. Breg is one of the major types of immune regulatory cells in the body. By releasing IL-10^16^ or transforming growth factor (TGF)-β^17^, Bregs suppress skewed immune responses; such as inhibit food antigen-related inflammation^18^.

We found a low level of allergen-specific IgE was detected in the sera of healthy subjects. These healthy subjects did not have allergic disease history. Thus, the production of allergen-specific IgE is not the sole factor to initiate allergic diseases.

In summary, the present data show that a portion of UC patients has skewed antigen-specific Th2 polarization. Treatment with SIT and Cb can regulate the skewed immune response and alleviate UC clinical symptoms.

## Materials and Methods

### Human Subjects

UC patients with or without food allergy (FA) were recruited into this study. The diagnosis of UC was performed by the physicians based on our routine UC diagnostic procedures and published data^19^. The diagnosis of FA was performed based on the FA history, skin prick tests and serum specific IgE. The demographic data of the UC patients are presented in Table 2. Healthy volunteers were also recruited as healthy controls who did not have FA and IBD history. This study was approved by the Human Ethic Committee at Fujian Medical University. The study was carried out in “accordance” with the approved guidelines. An informed written consent was obtained from each human subject.

### Skin Prick Tests (SPT)

The SPT was performed in our clinic using glycerinated food extracts (Greer Company; Taibei, China). Food extracts included cow’s milk, eggs, walnuts, hazelnuts, peanuts, soy, fish and shrimp. Histamine phosphate (1 mg/ml) was used as a positive control. Saline was used as a negative control. SPT was defined as positive when the greatest diameter of the wheal was at least 3 mm larger than a negative control at 15 min. The SPT results are presented in Table 2.

### Collection of peripheral blood samples and immune cell isolation

Peripheral blood samples were collected from each subject via ulnar vein puncture. The peripheral blood mononuclear cells (PBMC) were isolated from the blood samples by gradient density centrifugation. PBMCs were cultured in RPMI1640 medium supplemented with 10% fetal bovine serum, 100 U/ml penicillin, 0.1 mg/ml streptomycin and 2 mM L-glutamine. A portion of the PBMCs was processed for isolating CD4^+^ CD25^−^ T cells and dendritic cells (DC) by magnetic cell sorting with commercial reagent kits (Miltenyi Biotech; San Diego, CA) following the manufacturer’s instructions. The purity of the isolated cells was greater than 96% as assessed by flow cytometry. The sera were collected for further analysis.

### Challenge of PBMCs with specific food antigens in the culture

PBMCs (10^6^ cells/ml) were cultured in the presence of the specific food antigens (5 μg/ml) for 3 days. The supernatant was collected for the analyses of specific IgE levels (using an ImmunoCAP 250 system; Phadia, Uppsala, Sweden) and the levels of IL-4, IL-13, IFN-γ, TNF-α [by enzyme-linked immunoassay (ELISA) with commercial reagent kits (R&D Systems, Minneapolis, MN) following the manufacturer’s instructions].

### Flow cytometry

For the surface staining, cells were stained with the flouorochrome-labeled antibodies (or isotype IgG) at 4 °C for 30 min. For the intracellular staining, cells with or without surface staining were fixed with 2% paraformaldehyde for 2 h at 4 °C, and treated with 0.5% saponin for 30 min afterwards to increase the membrane permeability. The cells were then incubated with fluorochrome-labeled antibodies (or isotype IgG). The cells were analyzed with a flow cytometer. The data were processed with software Flowjo. Data of isotype IgG staining were used as a gating reference.

### Assessment of peripheral antigen-specific CD4^+^ T cells

CD4^+^ CD25^−^ T cells [labeled with CFSE (carboxyfluorescein succinimidyl ester; 1 μmol/ml) (Invitrogen; Carlsbad, CA) for 8 min at 37 °C] (10^5^ cells/well) and DCs (2 × 10^4^ cells/well) were cocultured for 3 days in the presence of specific food antigens (5 μg/ml) or BSA (5 μg/ml; using as an irrelevant allergen and as a negative control) and PMA (Phorbol 12-myristate 13-acetate; 20 ng/ml). The cells were collected at the end of the culture and analyzed by flow cytometry. The proliferating cells were regarded as the food antigen specific CD4^+^ T cells.

### Assessment of peripheral regulatory B cells (Breg)

The PBMCs were stained with anti-CD19 (FITC) and anti-IL-10 (PE) (BD Biosciences; Franklin Lakes, NJ) following published procedures^20^. The cells were analyzed by flow cytometry. The CD19^+^ IL-10^+^ cells were regarded as Bregs^21^.

### Assessment of UC symptoms

UC symptoms (mainly including rectal bleeding and stool frequency) were evaluated before and after the treatment based on the truncated Mayo score system^22^ (Table 3).

### Medication scores

The medication scores were recorded for each patient during the entire observation period. When necessary, the patients were allowed to be treated with mesalamine or/and to take prednisone based on published strategy^23^. The definition of medication score is presented in Table 4. The total medication scores were summarized by week and presented as averages of the one year before the treatment and the one year during the treatment.

### SIT

The SIT was carried FA-UC patients were treated with the sensitized foods once a week for 10 weeks. The weekly increment dosage is presented in Table 5. From the 11^th^ week to the 20^th^ week, the patients took the sensitized foods at 10 folds of the basic dose every two weeks. From the 21^st^ week to the end of the year, the patients took the sensitized foods at 10 folds of the basic dose once a month. The first treatment was carried out in our hospital.

### Treatment with probiotics

The Cb group patients were prescribed with the *C. butyricum* (Cb) capsule 420 mg, twice a day, throughout the observation period.

### Treatment of the placebo group

Patients were treated with sensitized foods and a capsule containing vehicle, but no Cb with the same dosage as the SIT group.

### Statistics

Data are presented as mean ± SD. Differences between two groups were determined by Student t test or ANOVA if more than two groups. p < 0.05 was set as a significant criterion.

## Additional Information

**How to cite this article**: Lan, B. *et al.* Specific immunotherapy plus Clostridium butyricum alleviates ulcerative colitis in patients with food allergy. *Sci. Rep.* **6**, 25587; doi: 10.1038/srep25587 (2016).

## Figures and Tables

**Table 1:** Positive SPT.

Milk	Egg	Walnut	Hazelnut	Peanut	Soy	Fish	Shrimp
35	21	8	6	11	5	8	8

The total number of patients was 80; some patients were sensitized to more than one antigen.

**Table 2:** Demographic data of UC patients.

Parameters	FA-UC (*n* = 80)	nFA-UC (*n* = 72)
Age, mean ± SD	35.8 ± 5.4	37.4 ± 4.7
Male	36 (45%)	29 (40.3%)
Diagnosis age (year)	22.6 ± 3.7	28.4 ± 4.1
Disease duration (year)	3.5 ± 2.4	4.3 ± 3.7
Proctitis	19 (23.8%)	15 (20.8%)
Left sided	10 (12.5%)	12 (16.7%)
Pancolitis	51 (63.8%)	45 (62.5%)
Mayo score		
Mild (%)	18 (22.5%)	10 (13.9%)
Moderate, *N* (%)	25 (31.3%)	20 (27.8%)
Severe, *N* (%)	37 (46.3%)	42 (58.3%)
Disease flares per year	1.88 ± 0.93	1.63 ± 0.59
Medication (%)		
Aminosalicylates	*56 (70%)	48 (66.7%)
Corticosteroids	36 (45%)	31 (43.1%)

^*^Some patients used more than one medicine.

**Table 3:** Truncated Mayo Score for rectal bleeding and stool frequency.

Rectal bleeding scale	
0	No blood seen
1	Streaks of blood with stool less than half of the time
2	Obvious blood with stool most of the time
3	Blood alone passed
**Stool frequency scale**	
0	Normal stool frequency per day
1	1–2 stools greater than normal per day
2	3–4 stools greater than normal per day
3	≥5 stools greater than normal per day

**Table 4:** Definition of medication scores.

Mesalamine	Scores	Prednisone	Scores
1 g	1	10 mg	1
2 g	2	20 mg	2
3 g	3	30 mg	3
4 g	4	40 mg	4

The dosage indicates daily dose.

**Table 5:** Antigen basic dose of SIT.

Milk	Egg	Walnut	Hazelnut	Peanut	Soy	Fish	Shrimp
10 ml	1/10 egg	3g	3g	3g	3g	3g	3g
